# Splitting the “Unsplittable”: Dissecting Resident and Infiltrating Macrophages in Experimental Autoimmune Encephalomyelitis

**DOI:** 10.3390/ijms18102072

**Published:** 2017-09-29

**Authors:** Tobias Koeniger, Stefanie Kuerten

**Affiliations:** 1 Institute of Anatomy and Cell Biology, University of Würzburg, D-97070 Würzburg, Germany; tobias.koeniger@uni-wuerzburg.de; 2 Institute of Anatomy and Cell Biology, Friedrich-Alexander University Erlangen-Nürnberg, D-91054 Erlangen, Germany

**Keywords:** CNS, distinction, experimental autoimmune encephalomyelitis, inflammation, macrophages, markers, microglia, monocytes

## Abstract

Macrophages predominate the inflammatory landscape within multiple sclerosis (MS) lesions, not only regarding cellularity but also with respect to the diverse functions this cell fraction provides during disease progression and remission. Researchers have been well aware of the fact that the macrophage pool during central nervous system (CNS) autoimmunity consists of a mixture of myeloid cells. Yet, separating these populations to define their unique contribution to disease pathology has long been challenging due to their similar marker expression. Sophisticated lineage tracing approaches as well as comprehensive transcriptome analysis have elevated our insight into macrophage biology to a new level enabling scientists to dissect the roles of resident (microglia and non-parenchymal macrophages) and infiltrating macrophages with unprecedented precision. To do so in an accurate way, researchers have to know their toolbox, which has been filled with diverse, discriminating approaches from decades of studying neuroinflammation in animal models. Every method has its own strengths and weaknesses, which will be addressed in this review. The focus will be on tools to manipulate and/or identify different macrophage subgroups within the injured murine CNS.

## 1. Introduction

Multiple sclerosis (MS) is a chronic inflammatory and neurodegenerative disease of the central nervous system (CNS) characterized by focal lesions of inflammation, demyelination, gliosis and axonal loss [1]. It is widely acknowledged that the autoimmune pathologies of MS and its most commonly used animal model, experimental autoimmune encephalomyelitis (EAE), are largely driven by self-reactive T cells [2]. However, many other immune and non-immune cells such as B cells, natural killer cells, neutrophils, macrophages, and astrocytes are significantly involved in the complex and highly variable processes that lead to demyelination and finally neurodegeneration [2,3].

Macrophages are the predominant inflammatory cells in active and chronic MS and EAE lesions [4,5,6,7]. They participate in disease progression and remission by providing a plethora of effector functions: macrophages present antigens to auto-reactive T cells and secrete cytokines, chemokines, free radicals, proteases as well as other mediators of tissue injury [8]. At the same time, they can facilitate remyelination by clearing myelin debris and promoting oligodendrocyte progenitor activity [9,10].

Depending on their origin, macrophages in CNS lesions can be categorized into at least two different groups: (1) blood-borne macrophages (moMΦ) that differentiate from monocytes invading the CNS after breakdown of the blood-brain barrier (BBB) and (2) resident macrophages, such as parenchymal microglia, that are already present in the CNS before disease onset. Accumulating evidence points to the fact that these populations fulfill unique roles in EAE and MS pathogenesis [11,12]. Sophisticated lineage tracing approaches as well as comprehensive transcriptome analysis have significantly advanced our knowledge on macrophage diversity over the last years, substantiating the notion that infiltrating and resident CNS macrophages are two separate entities with specialized functions and behaviors [13,14,15,16]. They have also given us a better understanding of the heterogeneity of myeloid cells within the CNS [17].

Due to the expression of a similar set of molecular markers, the discrimination of macrophage populations is still challenging once the cells intermingle under pathological conditions [7]. Respecting these difficulties, researchers often referred to macrophages in the injured CNS as a combined functional population termed “microglia/macrophage” [18,19,20]. Therefore important crosstalk between these populations as well as some of their functional divergence might have been poorly characterized or even completely overlooked. Over the last years a variety of tools has emerged to dissect the roles of myeloid cells in the inflamed murine CNS. In this review, we would like to discuss such novel as well as traditional approaches, while focusing on methods to genetically manipulate and/or identify infiltrating and resident macrophage populations in EAE.

## 2. A Novel View on the Mononuclear Phagocyte System

Monocytes and macrophages, including microglia, are parts of the mononuclear phagocyte system (MPS). Over many decades it was believed that every cell of the MPS was derived from a single source: the hematopoietic stem cells (HSCs) in the bone marrow (BM). Monocytes differentiating from HSCs were thought to be the common MPS precursors that disseminate through the bloodstream to constantly replenish macrophages all over the body, including the brain and spinal cord [21].

Only ten years ago, microglia were shown to self-renew inside the adult CNS without any contribution from circulating progenitors [22,23]. More importantly, monocytes do not even integrate into the microglia pool after they have been recruited into the injured CNS [24]. In accordance with the immune privilege of the CNS, these findings placed microglia in an extraordinary position compared to all other tissue-resident macrophages. Only a couple of years later, this view gained further support as the origin of microglia was finally elucidated: primitive macrophages that develop from erythro-myeloid precursors (EMP) in the yolk sac seed the mouse brain early during embryogenesis and differentiate into microglia [16,25,26,27,28].

However, this was only the beginning of a revolution in the macrophage field, in which HSCs and monocytes rapidly lost their central roles within the mononuclear phagocyte system (MPS). Today we know that most resident macrophage populations descend from embryonic progenitors and are able to self-renew independently of monocyte input, even if the extent seems to vary among tissues [29]. HSC-derived monocytes apparently only leave the bloodstream and differentiate into macrophages when the local self-renewal capacity is insufficient to satisfy the demand for mononuclear phagocytes, e.g., when there is constant exposure to germs such as in the gut and dermis or in an inflammatory lesion [29]. Several studies have shown that macrophage populations all over the body are highly specialized. They exhibit distinct transcriptional signatures and epigenetic marks in correspondence to their tissue of residence [30,31]. These signatures might be shaped by a combination of developmental imprinting and environmental cues [32].

Nevertheless, microglia still constitute a unique macrophage lineage, as their specification and expansion are independent of the transcription factors Myb, Id2, Batf3, and Klf4, in contrast to all other myeloid cells [25,26]. Only recently it was shown that other non-parenchymal CNS macrophages, such as perivascular (pvMΦ) and meningeal macrophages (mMΦ), share a common ontogeny with parenchymal microglia and also self-renew locally [33]. Accordingly, it was found that the transcriptional signature of pvMΦ is similar to microglia rather than to peripheral macrophages [33]. The sole exchange with peripheral mononuclear phagocytes appears to take place in the choroid plexus, where macrophages (cpMΦ) are slowly replaced by monocytes [33]. Therefore, when trying to understand the diverse contributions of macrophages in MS and EAE, one has to consider not only moMΦ and microglia, but also pvMΦ, mMΦ and cpMΦ as potential individual players in autoimmunity.

## 3. Strategies to Distinguish Infiltrating and Resident Macrophages in the Inflamed CNS

There are several methods and strategies that can be employed to discern infiltrating from resident macrophages in the inflamed CNS. They are described in detail in the following section and summarized in Table 1 and Table 2.

### 3.1. Discrimination Based on Anatomical Location

Before resident and monocyte-derived macrophages merge into a mixed population in inflammatory CNS lesions, these cells are easily distinguishable as they occupy different anatomical locations in the healthy organism. CNS myeloid cells can be targeted via injection of lentiviral particles into the mouse or rat brain. This approach can be used to tag them with fluorescent reporters and/or to alter expression of specific genes [34,35]. Apart from this route of administration, specificity for CNS myeloid cells has been accomplished by placing the transduced genes under regulation of e.g., the CD11b promoter [35] or microRNA-9 (miR-9) [34]. This is necessary as lentiviral vectors unselectively integrate into all the different CNS cell types. While the CD11b promoter is active specifically in myeloid cells, the latter are the only CNS cells lacking miR-9, which abolishes expression of transgenic mRNA carrying miR-9 target sequences.

Ding et al. showed that intracerebroventricular virus injection targeted more than 80% of microglia and pvMΦ of the brain and spinal cord [35], whereas the intracranial route only led to transduction of microglia around the injection site [34]. As CNS lesions appear at random locations in EAE, the latter approach does not seem favorable in this setting. Moreover, while the CD11b promoter drives stable and strong expression in CNS macrophages, it is unclear whether miR-9-based targeting is able to permit transgene expression in transduced microglia under EAE conditions. Åkerblom et al. reported the expression of delivered green fluorescent protein (GFP) irrespective of the activation status. Yet miR-9 has been shown to participate in microglial activation [36] and might therefore also block transgene expression following certain activating stimuli.

In vivo transduction bears the advantage that targeting of various modifications can be achieved without the need for genetic mouse models. However, application of lentiviral vectors into the CNS is technically challenging, highly invasive, and immunogenic [37]. It might therefore even influence the course of subsequent EAE. Besides, retro-/lentiviral transduction comes along with the disadvantages of variation due to copy-number effects as well as random integration. The latter will affect non-macrophage cells as well, even if they do not express the transduced gene.

### 3.2. Discrimination Based on Cell Morphology and Ultrastructure

Microglia show a unique ramified morphology in the healthy adult CNS. They present themselves with slim branching processes stretching from a compact cell body [38]. In the case of an inflammatory insult however, activated microglia retract their processes and shift to an amoeboid morphology, while migrating towards the site of inflammation [39,40]. Here, monocytes may enter the CNS through the leaky BBB and differentiate into macrophages, which share a similar amoeboid morphology. It is therefore acknowledged that cell shape cannot be used as a criterion to distinguish these two populations in CNS inflammation.

While this might be true for conventional light microscopy applications, successful discrimination between microglia and monocyte-derived macrophages has been reported in EAE using serial block face scanning electron microscopy (SBF-EM) [41]. Microglia were shown to have larger cell volume and a higher number of primary processes than monocyte-derived macrophages, while the latter were also characterized by shorter, thicker mitochondria, bi-lobulated or irregular nuclei, and they frequently contained osmiophilic granules and microvilli [41]. It is noteworthy though that these results were validated using *Ccr2*^rfp/+^::*Cx3cr1*^gfp/+^ mice. This system itself has certain limitations regarding the identification of monocyte-derived macrophages, which will be discussed in the following section.

### 3.3. Discrimination Based on Marker Expression

Elucidating the different roles of monocyte-derived and resident macrophages in EAE as well as other CNS diseases has been hampered, because these cells both express frequently used microglia/macrophage markers, including fractalkine receptor (CX_3_CR1), CD11b, F4/80, ionized calcium-binding adapter molecule 1 (iba-1) and CD68 [7]. Still several marker-based strategies have been, and are still used to discriminate between these populations, which will be described in the following section. Recently, comprehensive transcriptome analysis of various macrophage populations identified numerous new markers that hold the potential to better define the CNS myeloid compartment in health and disease. The most promising of them will be discussed below.

#### 3.3.1. Monocyte Markers: CCR2 and Ly6C

Two types of monocytes exist, including classical monocytes (Ly6C^hi^, CCR2^+^, CX_3_CR1^lo^) and non-classical monocytes (Ly6C^lo^, CCR2^−^, CX_3_CR1^hi^) [7]. In EAE, only classical monocytes are recruited into the CNS in a CCR2-dependent manner [7,42]. During its passage through the circulation as well as after extravasation, this monocyte subset can be easily distinguished from CNS macrophages via the expression of CCR2 and Ly6C [7,43].

Many researchers have therefore used the *Ccr2*^RFP/+^ mouse model, which marks CCR2^+^ monocytes via red fluorescent protein (RFP) expression [44], arguing that the RFP label would identify monocyte-derived macrophages within the macrophage pool [41,45]. Similar claims were made for Ly6C [46]. However, during differentiation into macrophages, classical monocytes downregulate CCR2 and Ly6C to the levels found on CNS myeloid cells [7,47]. CCR2 or Ly6C in combination with e.g., CX_3_CR1 or iba-1 thus only identify cells that are currently in a transitional state between these two cell types. This phase seems to be exceptionally short in the CNS compared to other tissues [48,49] limiting the usefulness of CCR2 and Ly6C as moMΦ markers in EAE even further. In the injured CNS of e.g., *Ccr2*^rfp/+^::*Cx3cr1*^gfp/+^ mice, infiltrating classical monocytes as well as differentiating moMΦ can indeed be identified as RFP^+^ GFP^lo^. However, as soon as these cells have reached the mature CCR2^−^ CX_3_CR1^+^ state, they have also shifted reporter expression and again become indistinguishable from resident CNS macrophages, which are RFP^−^ GFP^+^.

Ly6C- or CCR2-based lineage tracing systems, such as the *Ccr2*^CreERT2^ line [50] crossed to an inducible reporter strain may solve the problem of marker loss during differentiation. Here, tamoxifen-induced Cre recombinase could stably activate the expression of a reporter gene in CCR2^+^ cells to also label all CCR2^-^ macrophage progeny. This system can further be used to introduce genetic modifications specifically into monocytes and moMΦ [50]. Yet, these modifications would also affect NK cells and certain T lymphocytes, which express CCR2 [44,51]. Moreover, the induced recombination only targets CCR2^+^ monocytes that are circulating in the bloodstream or differentiating into macrophages at a given time point. As monocytes have a short half-life (~20 h) in the circulation [52], tamoxifen treatment has to be applied carefully to ensure optimal targeting of the moMΦ compartment according to the desired experimental setup. At the same time, this allows relatively precise, temporary control of the genetic manipulation within the monocyte pool, in contrast to e.g., regular BM transfer models.

#### 3.3.2. The Microglia CD45^lo/int^ Phenotype

A popular approach used to identify microglia is based on their uniquely low expression of the tyrosine phosphatase CD45 [53]. Following dissociation of CNS tissue, single cell suspensions can be analyzed via flow cytometry. Here, microglia present themselves as CD11b^+^ CD45^int^ and can be clearly separated from other macrophages, including mMΦ, pvMΦ and cpMΦ, which are CD11b^+^ CD45^hi^. On top of this approach pvMΦ, mMΦ and cpMΦ can further be identified via the expression of the endocytic pattern-recognition receptor CD206 (also known as mannose receptor C type 1 or MRC1) [33,54]. Moreover, mainly pvMΦ and some mMΦ and cpMΦ have been shown to express the scavenger receptor CD163 [55,56,57,58].

While this simple method works well under healthy conditions, its specificity is lost during CNS inflammation. Undisputedly, monocyte-derived macrophages are found within the CD11b^+^ CD45^hi^ population. However, based on these two markers alone, they cannot be distinguished from their monocyte precursors (CD11b^+^ CD45^hi^ Ly6C^+^ CX_3_CR1^lo^) as well as from neutrophils (CD11b^+^ CD45^hi^ Ly6G^+^) and monocyte-derived dendritic cells, which also gain entry into the CNS after breach of the BBB [7]. It has also been mostly ignored that with this strategy, monocyte-derived macrophages in the inflamed CNS cannot be separated from non-parenchymal CNS macrophages, which at least in part seem to be related to microglia despite their CD11b^+^ CD45^hi^ profile [33].

It was suggested that CD206 could be a useful marker to differentiate pvMΦ, mMΦ and cpMΦ from CD11b^+^ CD45^hi^ moMΦ in the course of CNS inflammation [54]. However, this molecule has long been regarded as a marker for the so-called “M2” phenotype of macrophages [59,60]. Thus, it is conceivable that other macrophage subsets upregulate CD206 under certain circumstances. The same applies to CD163 [60], which has already been demonstrated on activated microglia [61,62,63,64] as well as monocytes/macrophages [65].

Apart from the obvious flaws of the CD45-based separation strategy, there is also the notion that the clear line between CD45^hi^ macrophages and CD45^int^ microglia might start to blur during inflammation, as activated microglia in different species have been reported to upregulate CD45 [66,67,68,69,70,71]. Recently, O’Koren and colleagues have disproven this concern using an elegant *Cx3cr1*^CreER^-based lineage tracing approach, which will be discussed in a following section [49]. They show that the expression of CD45, as well as of other markers that have been thought to assimilate their expression to the level of moMΦ, is only marginally altered on activated microglia, therefore allowing a distinction between microglia and moMΦ even in the setting of inflammation. They characterized microglia as CD11b^+^ Ly6C^−^ Ly6G^−^ CD64^+^ CD45^lo^ CD11c^lo^ F4/80^lo^ I-A/I-E^−^ (commonly known as major histocompatibility complex class II), while moMΦ were CD11b^+^ Ly6C^−^ Ly6G^−^ CD64^+^ CD45^+^ CD11c^+^ F4/80^+^ I-A/I-E^+^.

This strategy is clearly more preferable than the basic two-marker approach, as it excludes non-macrophage cell types from the analysis. It is hitherto questionable whether it is able to distinguish between moMΦ and CNS-resident CD45^hi^ macrophages, which are for example also F4/80^+^ and I-A/I-E^+^ [17]. More importantly, it has only been tested in the context of light-induced retinal injury [49]. Whether this approach is applicable to EAE remains to be shown. In previous attempts to improve the CD11b/CD45 strategy, CD39 [46] and CD44 [72] were suggested as additional microglia and monocyte/moMΦ markers, respectively. However, these markers were only validated using other marker-based approaches (Ly6C, CD45) during CNS inflammation [46,72]. Their specificity is therefore still insufficiently resolved.

#### 3.3.3. New Microglial Markers

In-depth transcriptomic analysis of different macrophage populations recently identified several new markers that were termed microglia-specific, including *Sall1*, *Tmem119*, *HexB*, *Tgfbr*, *Olfml3*, *P2ry12*, *P2ry13*, *Gpr34*, *Fcrls*, and *Siglech* [14,30,31,45,73,74]. However, the significance of some of these studies with respect to EAE is limited by the choice of compared macrophage populations [73], the lack of an exhaustive comparison to other immune cells [73,74] or the missing evaluation under inflammatory conditions [30,73]. Moreover, all those studies used bulk RNA for transcriptome analysis and did not include non-parenchymal CNS macrophages. Other single-cell RNA sequencing studies have revealed highly cell type-specific markers for microglia (*P2ry12*, *Gpr34*, *Cd83* and *HexB*) and pvMΦ (*CD163*, *Hpgd*, *Mrc1*, *Slc40a1* and *F13a1*) in the steady state [17]. Among those markers stated above, Sall1 and Tmem119 have been extensively studied and will therefore be discussed here in more detail.

TMEM119 is a transmembrane protein of unknown function and has been characterized as a microglial marker in mice and humans [45,75]. It is developmentally regulated and seems to be expressed by microglia as they mature, starting at postnatal day 14 in mice. *Tmem119* mRNA is expressed solely by parenchymal microglia in the CNS, but by no other neural or glial cell type. Importantly, also pvMΦ, mMΦ, and cpMΦ lack *Tmem119* mRNA. Furthermore, it was absent at all peripheral sites tested, including the liver, BM, thymus, blood, spleen and peripheral nerve even in the context of inflammation and injury [45].

In contrast to Tmem119, expression of the zinc-finger transcription factor Sall1 was so far only demonstrated in murine microglia [15,31,76]. *Sall1* is not expressed in CD45^+^ hematopoietic cells outside the CNS, but in CD45^−^ cells in liver, kidney and heart. Within the CNS, microglia are the only cell type that expresses *Sall1*, as this factor was not detected in neuronal progenitors, neurons, astrocytes and oligodendrocytes. As for Tmem119, cpMΦ lack *Sall1* expression, while a small subpopulation (~5%) of pvMΦ and mMΦ was shown to be *Sall1* positive [15].

Sall1 has been shown to control microglia identity in vivo by silencing an inflammatory program [15]. It is therefore important to note that intraperitoneal injection of LPS led to the downregulation of *Sall1* in microglia [15]. As Sall1 seems to control the expression of other signature genes such as *P2ry12*, which were downregulated after conditional knock-out of *Sall1* [15], this raises the question whether activated microglia lose the expression of most of the newly identified signature genes during CNS inflammation. Accordingly, *P2ry12* has already been shown to become downregulated upon microglial activation [39]. Other investigators have also reported the downregulation of microglia signature transcripts following LPS injection [45] as well as in mouse models of amyotrophic lateral sclerosis (ALS) [74] and Alzheimer’s disease (AD) [77]. However, Bennet and colleagues stated that activated microglia could still be specifically labeled with anti-TMEM119 antibody despite reduced *Tmem119* mRNA levels [45]. It remains to be shown if this is also the case for other signature markers.

The potential of *P2ry12*, *Fcrls*, *Tmem119*, and *Sall1* to discriminate microglia from monocyte-derived macrophages has already been tested in models of CNS injury as well as EAE [14,15,45], though these studies relied solely on CCR2 reporter mice as well as BM chimeras (which will be discussed later in more detail), to which certain limitations apply. As transfer of labeled BM for instance typically does not lead to complete chimerism, there is always a small population of non-labeled moMΦ present in the inflamed tissue. Sorting labeled cells from the CNS can still yield a pure moMΦ population, and it was unequivocally shown that these infiltrated cells indeed do not acquire the expression of microglia signature genes [14,15,45]. Conversely, in case a fraction of microglia at the lesion site loses expression of signature markers, the latter will always be misinterpreted as incomplete chimerism in immunohistochemical studies, especially as moMΦ outnumber microglia in lesions during EAE progression [24,72]. In flow cytometry or pooled RNA analysis, this population might even go unnoticed. So while it can be acknowledged that invading moMΦ do not express microglial signature genes and microglia do not lose signature gene expression on a global scale during CNS inflammation, it cannot be excluded that highly activated microglia in the lesion center might strongly shift their expressional profile towards an inflammatory phenotype making them indistinguishable from their monocyte-derived counterparts.

Even if these newly discovered signature genes lose some of their specificity under inflammatory conditions, they still allow an unprecedented definition of microglia in the healthy organism. In some cases, one marker alone seems sufficient for this definition, which opens up opportunities for conditional gene targeting. In the *Sall1*^CreER^ mouse line, tamoxifen pulsing leads to recombination of floxed target genes in 90% of microglia [15,78]. As microglia are long-lasting and potentially self-renew via division of differentiated cells, this recombination should be stably maintained within the microglia population [13,79]. Crossing these mice to e.g., a *ROSA26*^-stop-YFP^ reporter strain [80] would permit the specific labeling of microglia before the onset of CNS inflammation, while crossing them to e.g., conditional knock-out strains enables targeted manipulation of microglia [15].

### 3.4. Discrimination Based on Self-Renewal and Turnover

Whereas microglia as well as pvMΦ and mMΦ already reside and slowly self-renew inside the healthy CNS, monocytes are constantly produced from HSCs in the BM and first have to travel through the bloodstream to enter the CNS in the event of injury. These differences in the biology of CNS macrophages and monocytes can be exploited experimentally to discriminate between them and their progeny within CNS lesions, which will be discussed in detail in this section.

#### 3.4.1. Bone Marrow Chimeras

Stable labeling of blood cells including monocytes can be achieved by exchanging their BM source with BM cells that have been collected e.g., from a ubiquitous GFP reporter mouse. Upon transfer of such cells into a myeloablated wildtype recipient, over 90% of circulating monocytes will usually be of donor origin, which can be easily distinguished from tissue macrophages by reporter expression [81,82]. It has been shown that the method for myeloablation has to be chosen carefully. Widely used whole body irradiation damages the BBB [83,84] and promotes artificial engraftment of transferred hematopoietic progenitors, which normally are not present in the bloodstream, into the CNS, where they can differentiate into macrophages [13,24,85]. This engraftment preferentially takes place around blood vessels and within the leptomeninges [86,87,88], where also EAE lesions develop [89,90]. Shielding the head during irradiation can avoid this problem in the brain [23], but not in the spinal cord, where much of EAE pathogenesis takes place [90,91]. It is controversial whether replacement of irradiation with the alkylating agent busulfan as myoablative agent prevents artificial CNS engraftment [81,82,85,92]. This might in fact depend on the exact treatment regimen. Alternatively, the related drug treosulfan has been suggested to prevent CNS engraftment after BM transplantation due to its lower CNS penetration [85].

BM transfer provides the advantage that modifications of blood cells can easily be introduced by using donor cells from knock-out or transgenic mice [23,42,93]. Where the desired models are not available, BM cells can be transduced with retro- or lentiviral vectors ex vivo, before transfer into the recipient [94]. As mentioned earlier, this is associated with the disadvantages of random integration and copy number effects. The development of protocols for the differentiation of murine HSCs from induced pluripotent stem cells in combination with Crispr/Cas9 technology might be able to circumvent this issue in the near future [95].

#### 3.4.2. Parabiosis

Parabiosis enables partial labeling of blood cells, including monocytes. In this approach, the blood circulation of two syngeneic mice is connected surgically. Using one wildtype recipient and one transgenic donor animal, in which e.g., GFP is ubiquitously expressed as a reporter, 40–50% of circulating blood cells in the wildtype animal will be GFP^+^, while myeloid cells within the CNS remain GFP^−^. When inducing CNS injury in the recipient mice, infiltrating cells can be tracked via the GFP label [22,24].

Apart from the technical challenge, a major drawback of this model is the low level of chimerism that leads to an underestimation of infiltrating cells [81]. Ajami and colleagues reported that the rate of general chimerism can be increased to 80% by lethal irradiation of the recipient [22]. In this model, the ablated BM of the recipient is populated by physiologically circulating HSCs from the donor [96,97], which has been shielded from irradiation. Following this physiologic HSC transfer, joined mice can be separated again, yielding a BM transfer in the absence of artificial CNS engraftment [24]. However, the observed chimerism (>60% after separation) was still relatively low in comparison to BM transplantation [24]. More importantly, the exchange of blood-borne cells or proteins between parabiotic mice is directly related to their individual half-lives in the circulation [96,97,98]. This is why B and T cells reach equilibrium in the blood, in contrast to cells with high turnover such as polymorphonuclear leukocytes (~40% chimerism in the recipient) and Ly6C^hi^ monocytes (~30% chimerism in the recipient) [99]. Finally, parabionts may experience a higher stress level, which is known to dampen EAE progression [100,101]. This was already confirmed in the parabiotic EAE studies of Ajami and colleagues [24].

#### 3.4.3. *Cx3cr1*-Based Lineage Tracing

Currently, the most elegant way to discriminate CNS myeloid cells from moMΦ is based on the transgenic *Cx3cr1*^CreER^ mouse line, which was generated in parallel by two groups [52,102]. Here, tamoxifen-inducible Cre recombinase is knocked into the fractalkine receptor (CX_3_CR1) gene locus. Crossing these mice with e.g., a *ROSA26*^-stop-YFP^ reporter strain [80] enables the induction of stable yellow fluorescent protein (YFP) expression in CX_3_CR1^+^ cells via tamoxifen pulsing.

The *Cx3cr1* promoter is active not only in CNS myeloid cells, but also in circulating monocytes and other peripheral mononuclear phagocytes, including macrophages and dendritic cells as well as myeloid progenitors in the BM [103,104]. Therefore, all of these cells are labeled after the initial tamoxifen pulse. Specificity is achieved based on differences in how these cell populations are maintained over time. While CNS myeloid cells are long-lasting and self-renew from a labeled CX_3_CR1^+^ population within the CNS [13,33], monocytes rapidly turn over in the circulation and have to be constantly replenished by HSCs. However, HSCs do not express CX_3_CR1 and are thus not affected by the tamoxifen-induced recombination. After a four-week wash-out period, no more YFP^+^ cells can be found in the circulation, whereas resident macrophages inside the CNS retain the YFP label [105]. In health and disease, resident cells can now be distinguished from monocyte-derived macrophages via the expression of the induced reporter [13,33,49,105]. As long as recombination is induced at least four weeks in advance of CNS injury, infiltrating monocytes and their macrophage progeny will not acquire the YFP label, even when their marker signature including CX_3_CR1 becomes indistinguishable from resident CNS macrophages. This is why this strategy is preferable over direct fluorescent reporter models such as *Cx3cr1*^gfp/+^ or *Ccr2*^RFP/+^. Further advantages of this approach are its non-invasive character, relatively low technical effort as well as its high specificity. High labeling efficiencies ranging from 80% to 99% of microglia can be achieved [13,33,102,105], which might probably depend on the exact protocol used for Cre induction. Still, this method cannot discriminate microglia from other CNS-resident myeloid cells. Perivascular macrophages are equally labeled in this approach, while Goldmann and colleagues reported a reduced labelling of mMΦ (40–50% YFP^+^). Although cpMΦ were shown to be slowly replaced by circulating monocytes, about 40% of these cells retained the YFP label after more than 40 weeks post induction [33].

It has been shown that the *Cx3cr1*^CreER^ line represents an excellent system to decipher the role of certain factors specifically in CNS myeloid cells during inflammation, e.g., via conditional knock-out [93,105]. Nevertheless, while it achieves clear differentiation between resident and infiltrating macrophages in the CNS, one has to keep in mind that even after the wash-out some peripheral CX_3_CR1^+^ populations will also be targeted [52]. More importantly, gradual tamoxifen-independent recombination in microglia was observed in one of the generated *Cx3cr1*^CreER^ mouse lines [106]. While this did not impair the cellular specificity of this model [102], it obviously abolishes timed recombination. Hence, introduced manipulations can already affect CNS development, in which microglia are essentially involved [107].

## 4. Conclusions

In EAE, many attempts have been made to distinguish monocyte-derived from CNS-resident macrophages. These ambitious efforts have been rewarded with accumulating evidence that supports the specialized roles of these cells during disease progression and remission. However, the discrimination and selective targeting of different myeloid populations during EAE remains a significant task. While immunological markers and reporter lines can be used to obtain fast results, these are usually ambiguous and have to be confirmed by BM transplantation or genetic models. Newly identified microglial markers, Cre-based targeting systems as well as growing insight into the myeloid diversity within the CNS bear the potential to revolutionize macrophage research in EAE. Nonetheless, every experimental model holds its innate limitations. Scientists should properly consider these when interpreting previous data and designing future studies.

## Figures and Tables

**Table 1:** Methods to discriminate infiltrating from resident CNS macrophages.

Approach	Discrimination	Principle	Limitations	References ^†^
Ultrastructure	MG ↔ moMΦ	MG show larger cell volume and higher number of primary processes.	Elaborate, specificity problematic, no data on non-parenchymal CNS macrophages.	[41]
Monocyte markers	moMΦ ↔ {MG, pvMΦ, mMΦ, (cpMΦ) *}	moMΦ retain the monocyte-specific expression of Ly6C and CCR2 on their surface.	Only identifies cells in the short process of differentiation towards moMΦ.	[44,46]
Differential surface marker expression	MG ↔ {moMΦ, pvMΦ, mMΦ, cpMΦ}	MG display lower surface expression of e.g., CD45 and F4/80.	No clear-cut discrimination due to marker upregulation in activated MG.	[49]
Microglia signature markers	MG ↔ {moMΦ, pvMΦ, mMΦ, cpMΦ}	MG show stable cell type-specific expression.	At least some markers are downregulated/lost during activation.	[14,15,45]

MG: microglia; moMΦ: monocyte-derived macrophage; pvMΦ: perivascular macrophage; mMΦ: meningeal macrophage; cpMΦ: choroid plexus macrophage; ( ) * applies only partially; ^†^ exemplary publications in which the respective approach has been utilized are referenced to.

**Table 2:** Methods to distinguish and genetically modify infiltrating and resident CNS macrophages.

Approach	Discrimination	Principle	Limitations	References ^†^
In vivo transduction	{MG, pvMΦ} ↔ moMΦ	Lentiviral particles transduce all CNS cell types after i.c.v. injection. Transgene expression is regulated e.g., via a macrophage-specific promoter.	Technically challenging, invasive, immunogenic, variation due to random integration and copy-number effects, system to regulate transgene expression has to be chosen carefully.	[34,35]
Bone marrow chimeras	moMΦ ↔ {MG, pvMΦ, mMΦ, (cpMΦ) *}	HSC source of blood monocytes is replaced with labeled/modified HSCs.	Careless selection and control of myeloablation may lead to artificial engraftment of BM-cells in the CNS.	[23]
Parabiosis	moMΦ ↔ {MG, pvMΦ, mMΦ, (cpMΦ) *}	Monocytes from a different labeled/modified HSC source are continuously introduced into the bloodstream.	Technically challenging, low chimerism, increased stress dampens EAE progression in parabiotic animals.	[24]
*Ccr2*^CreER^ line	moMΦ ↔ {MG, pvMΦ, mMΦ, (cpMΦ) *}	Label/modification is induced in CCR2^+^ circulating monocytes prior to their differentiation into moMΦ.	Also targets NK cells and some T cells.	[50]
*Sall1*^CreER^ line	MG ↔ {moMΦ, pvMΦ, mMΦ, cpMΦ}	Label/modification is induced in Sall1^+^ microglia.	Recombination can only be induced with high specificity prior to MG activation, unspecific targeting of non-hematopoietic cells in liver, kidney and heart.	[15]
*Cx3cr1*^CreER^ line	{MG, pvMΦ, mMΦ, (cpMΦ) *} ↔ moMΦ	Recombination is induced in CX_3_CR1^+^ cells. Long-lived & self-renewing CX_3_CR1^+^ CNS macrophages retain the label/modification, while short-lived monocytes are replenished from CX_3_CR1^−^ HSCs not carrying the recombination.	Spontaneous recombination in one mouse line reported, relatively low recombination in mMΦ (40–50%).	[52,102]

MG: microglia; moMΦ: monocyte-derived macrophage; pvMΦ: perivascular macrophage; mMΦ: meningeal macrophage; cpMΦ: choroid plexus macrophage; ( ) * applies only partially; ^†^ exemplary publications in which the respective approach has been utilized are referenced to.

## Abbreviations

AD	Alzheimer’s disease
ALS	Amyotrophic lateral sclerosis
Batf3	Basic leucine zipper transcription factor, ATF-like 3
BBB	Blood-brain barrier
BM	Bone marrow
CCR2	C-C chemokine receptor type 2
CNS	Central nervous system
cpMφ	Choroid plexus macrophage
EAE	Experimental autoimmune encephalomyelitis
EMP	Erythro-myeloid precursor
GFP	Green fluorescent protein
HSC	Hematopoietic stem cell
Id2	Inhibitor of DNA binding 2
Klf4	Kruppel-like factor 4
LPS	Lipopolysaccharide
Ly6C	Lymphocyte Ag 6C
mMφ	Meningeal macrophage
moMφ	Monocyte-derived macrophage
MPS	Mononuclear phagocyte system
MS	Multiple Sclerosis
Myb	Myeloblastosis
pvMφ	Perivascular macrophage
RFP	Red fluorescent protein
SBF-EM	Serial block face scanning electron microscopy
YFP	Yellow fluorescent protein
